# Interfacial Phenomena Governing Performance of Graphene Electrodes in Aqueous Electrolyte

**DOI:** 10.1021/acs.nanolett.4c01808

**Published:** 2024-09-04

**Authors:** Marta Delgà-Fernández, Alejandro Toral-Lopez, Anton Guimerà-Brunet, A. Pablo Pérez-Marín, Enrique G. Marin, Andrés Godoy, Jose A. Garrido, Elena del Corro

**Affiliations:** † Catalan Institute of Nanoscience and Nanotechnology (ICN2), CSIC and BIST, 08193 Bellaterra, Spain; ‡ Pervasive Electronics Advanced Research Laboratory (PEARL), Department of Electronics and Computer Technology, University of Granada, 18071 Granada, Spain; § Institut de Microelectrònica de Barcelona (IMB-CNM), CSIC, Esfera UAB, 08193 Bellaterra, Spain; ∥ Centro de Investigación Biomédica en Red en Bioingeniería, Biomateriales y Nanomedicina (CIBER-BBN), 28029 Madrid, Spain; ⊥ ICREA, 08010 Barcelona, Spain

**Keywords:** graphene, interfacial phenomena, electrical double layer, water intercalation

## Abstract

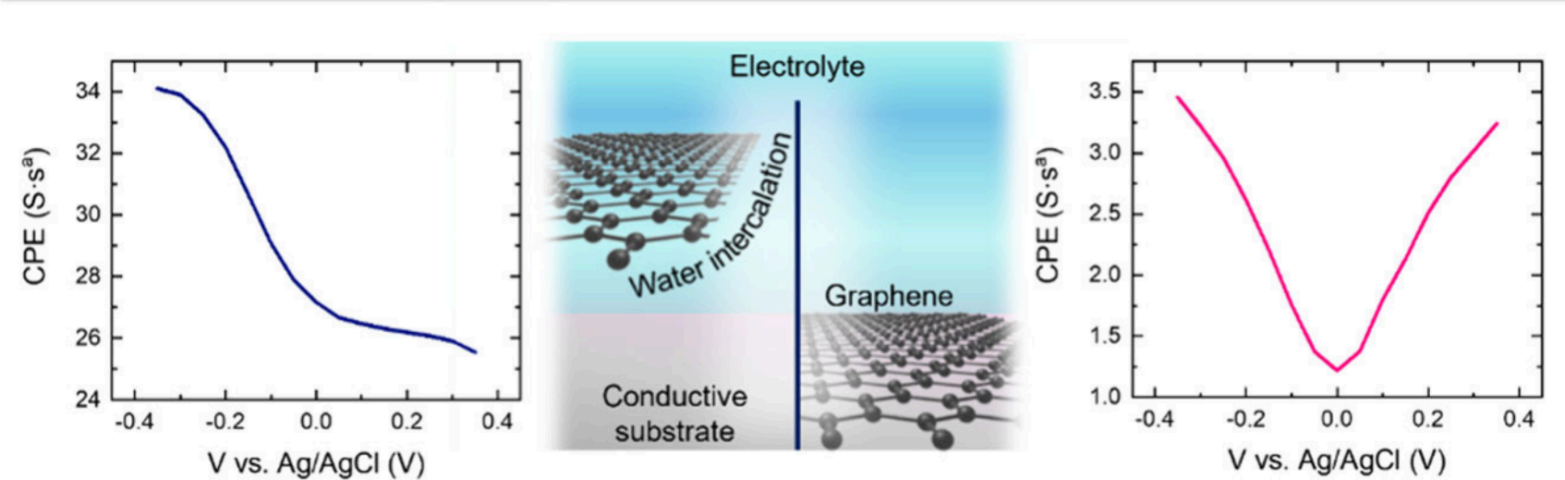

There is evidence of the presence of intercalated water between graphene and the substrate in electronic devices. However, a proper understanding of the impact of this phenomenon, which causes important limitations for the optimization of graphene-based devices operating in aqueous electrolytes, is missing. We used graphene-based electrodes on insulating and conducting substrates to evaluate the impact of intercalated water by combining experimental techniques with numerical simulations. Results show that the capacitance of the conductive substrate/graphene electrodes is significantly higher than that of the insulating substrate/graphene ones. Meanwhile, Raman spectroscopy demonstrates that graphene charge modulation with the applied potential is independent of the substrate conductivity. We found that this intriguing behavior is influenced by the water intercalation phenomena and governed by the substrate conductive nature. This work contributes to the understanding of the electric response of graphene-based devices in an aqueous environment and of the methods to measure and model it.

Since graphene first experimental isolation in 2004,^1^ numerous studies have reported on its unique combination of electronic, mechanical, chemical and optical properties. Profiting from this basic research, graphene-based electronic devices are nowadays investigated for a wide range of applications. Particularly, graphene electronics are being actively explored in biosensing and biomedicine, for instance, in applications including pharmacology,^2,3^ diagnosis^4,5^ and neural implants.^6,7^ For these applications, graphene is aimed to work in contact with an aqueous media (typically an electrolyte) while maintaining its structure and enabling long-term functionality and stability.^8^ Several works have highlighted the importance of knowing how an electrolyte in contact with graphene may impact its electronic properties and, subsequently, its applications.^9,10^ Numerical simulations and experiments have revealed that the electrolyte ionic composition and concentration determine the ionic adsorption and the properties of water in the direct vicinity of the graphene surface,^11−13^ thus directly affecting the electrical double layer (EDL) formed at the graphene–electrolyte interface. However, it is not only the water above graphene that has to be considered to properly understand this EDL, it is known that water can intercalate between graphene and the substrate influencing their electrical coupling.^14,15^ Confined water underneath graphene may be present due to the transfer process of graphene to the substrate.^16,17^ Furthermore, considering situations in which graphene devices are immersed in an aqueous electrolyte, for instance, during the fabrication of the devices or during their use in biomedical applications, water intercalation can also occur^18^ and depends on the graphene quality. Therefore, even performing a water-free transfer process, water can intercalate while the device is being used.^19^ Understanding and controlling the role of water intercalation in such devices is of utmost importance to achieve the high performance of graphene-based technologies in the biomedical field. The physical nature of the confined water differs from that of bulk water, and it is partially governed by the chemical and electrical characteristics of the substrate. Thus, the properties of the graphene substrate not only have a great influence on the electronic properties of this bidimensional material and on the formation of the EDL,^20,21^ but also on the performance of electronic devices operating in aqueous conditions.

Here, we study the interfacial substrate/graphene/electrolyte phenomena combining potentiostatic electrochemical impedance spectroscopy (PEIS), Raman spectroelectrochemistry and numerical simulations. To study the impact of the interfacial water, we compared pyrex/graphene with ITO/graphene electrodes. Both substrates are oxides yet with very different conductivity; in this way, we can study the impact of confined water on the measured electrical properties. For the pyrex/graphene system, impedance spectroscopy reveals an interfacial capacitance around 1.5 μF/cm^2^, with the expected symmetric modulation with the applied voltage.^22^ However, for the ITO/graphene configuration, we register an 18-fold increase of the interfacial capacitance and no symmetric modulation. Raman spectroscopy measurements show almost identical charge modulation in the graphene layer for both substrates, which, in principle, would not be expected from the capacitance results. These observations indicate that, while graphene charge modulation is possible regardless of the substrate conductivity, intercalated water governs the electrical response of the whole structure in aqueous media. We used numerical simulations to better understand this phenomenon. In this work, we provide a complete analysis of the system, including all the involved elements and their interfaces, thus contributing to advance toward an optimization of graphene-based electronic devices.

Samples were prepared as detailed in the Methodology (SI). The observation and analysis of interfacial electrostatic interactions among the elements of the substrate/graphene/electrolyte system are enabled by PEIS. In our case, the applied voltage range was selected within the electrochemical potential window of graphene (see SI), as described in Figure 1a. Figure 1b shows the Bode representation of the PEIS of graphene on pyrex and ITO, depicting module (|*Z*|) and phase of impedance as a function of frequency at three selected voltages (complete data set in SI). The fitted region of the experimental results considers the nonfaradaic regime, that is, where no electrochemical reactions are present,^23^ from 40 Hz to 4 × 10^4^ Hz. Fitting of the PEIS data was conducted using a distributed elements model represented by the equivalent circuit depicted in Figure 1c, where *R*_c_ includes the contribution of the contact resistance and the electrolyte resistance and *R*_sh_ corresponds to the graphene sheet resistance, defined as a distributed element.^24^ A constant phase element (CPE) has been considered to model the EDL formed at the graphene–electrolyte interface.^25^ In our case, the “a” parameter of the CPE is close to 1 (0.9), confirming that capacitive behavior governs the impedance. *R*_subs_ refers to the resistive component of the substrate, which has been approximated to ∞ and thus not considered for the fitting. SI provides a more detailed description of the fitting model.

**Figure 1:**
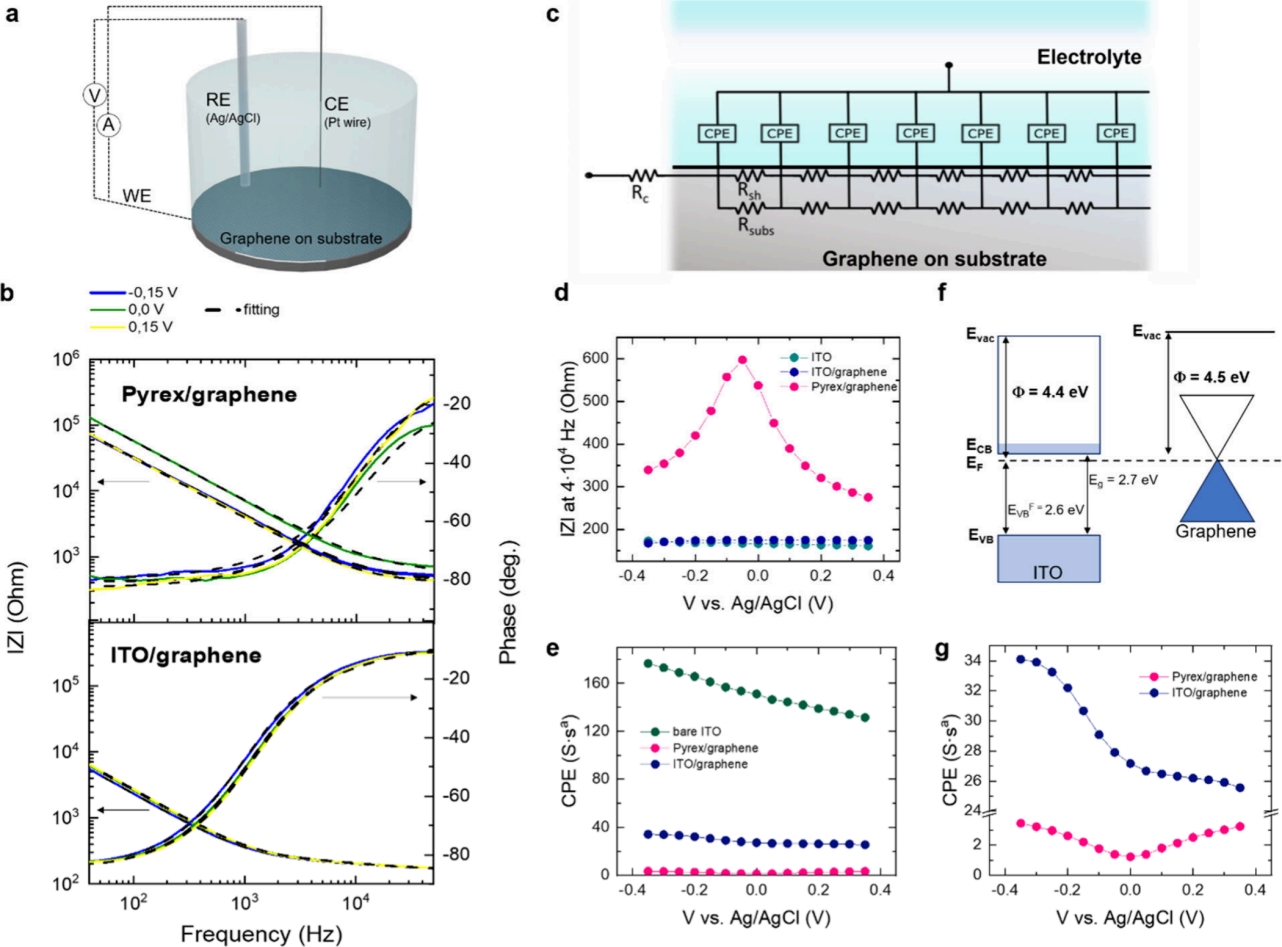
(a) Schematic of the PEIS setup. (b) PEIS Bode curves of graphene on pyrex and on ITO measured at 3 voltages (−0.15 V (blue), 0 V (green), and 0.15 V (yellow)). The fitting of the data performed by the model is represented with black dashed lines. (c) Electrolyte/graphene interface schematically represented with the equivalent circuit model used for the PEIS fitting. (d, e) |*Z*| extracted at 4 × 10^4^ Hz, and CPE modulation with V for the bare ITO, pyrex/graphene, and ITO/graphene electrode. Complete PEIS data set for bare ITO is provided in Figure S4. (f) Band diagram model of ITO and graphene. Energy levels are specified as the vacuum level *E*_vac_, the Fermi level *E*_F_, the conduction band minimum *E*_CB_ and the valence band maximum, *E*_VB_. *E*_VB_^F^ is extracted from the ultraviolet photoelectron spectroscopy (UPS) measurement shown in Figure S6. (g) Zoom-in of the CPE modulation with V for pyrex/graphene and ITO/graphene. Values in d, e and g are extracted from the fitting.

The Bode curves in Figure 1b reveal noticeable differences between the graphene electrodes on pyrex and on ITO. First, we observe a clear voltage dependence of |*Z*| for pyrex/graphene electrodes, which is less obvious in the case of ITO/graphene electrodes. In addition, |*Z*| is significantly different in the two types of electrodes, at both low and high frequency. The impedance of ITO/graphene is clearly lower than the impedance of the pyrex/graphene. In Figure 1d, we present the voltage dependence of |*Z*| at 4 × 10^4^ Hz, a frequency at which the impedance is mostly dominated by the resistive components of the equivalent circuit (Figure 1c.) The pyrex/graphene electrode shows the expected voltage dependence for the graphene *R*_sh_, an inverted V-shaped curve corresponding to the ambipolar nature of graphene,^24^ with a zero bandgap electronic structure that enables switching between electron and hole conductivity.^26^ The maximum of the curve is the point of minimum conductivity, corresponding to the charge neutrality point (CNP).^27^ In contrast, the impedance of the ITO/graphene electrode in the high frequency regime does not exhibit the characteristic voltage dependence of the graphene R_sh_; instead, the measured response resembles that of bare ITO electrodes (see SI for the complete PEIS data). At high frequencies, where impedance is governed by the resistive components, the low resistance of the ITO substrate dominates, and the contribution of the graphene R_sh_ is negligible.

In Figure 1e we show the CPE fitting values of the pyrex/graphene and ITO/graphene electrodes as a function of the applied voltage. For comparison, we also depicted the CPE of a bare ITO electrode. In Figure 1g, we zoom in on the CPE values for the ITO and pyrex graphene electrodes. The CPE of pyrex/graphene, which we directly assign to the interfacial capacitance of the graphene electrode, exhibits the expected V-shape response characteristic of the graphene-electrolyte interface, resulting from the series combination of graphene quantum capacitance and EDL capacitance. The measured magnitude of the interfacial capacitance, ∼1.5 S·s^a^ (“a” is the dispersion coefficient),^28^ is in good agreement with the reported values for graphene electrodes.^22,29^ However, for the ITO/graphene electrode, CPE values reach ∼27 S·s^a^ at 0.0 V. This is 18-fold higher than the value measured in pyrex/graphene electrodes and, at the same time, lower than the one for bare ITO electrodes, ∼140 S·s^a^, which is in good agreement with n-doped ITO (see Figure S7 for a more detailed analysis of the voltage-dependent interfacial impedance of the bare ITO electrodes and its relation with the doping level). Interestingly, our results reveal that the CPE of ITO/graphene does not exhibit symmetric behavior with respect to the applied voltage, in clear contrast to the case of pyrex/graphene. This could be explained by a nonefficient modulation of the graphene concentration of dopants when graphene is deposited on an n-type semiconductor as ITO. As can be inferred from the band diagram of Figure 1f, where the work function of ITO (Φ_ITO_) is slightly lower than the work function of graphene (Φ_graphene_),^30−32^ ITO should enable a more efficient modulation of holes in graphene.

To further understand the response of graphene electrodes on insulating and conductive substrates, we used Raman spectroelectrochemistry to directly measure the charge density at the graphene surface. Thanks to the strong electron–phonon coupling in graphene, Raman spectroscopy can be applied to measure its surface charge density.^33^

We performed Raman mapping of the graphene electrodes immersed in aqueous electrolyte while applying different voltages (−0.35 to 0.35 V vs Ag/AgCl). Figure 2a depicts two exemplary Raman maps of the frequency of the G band (ω_G_) for an ITO/graphene electrode collected at *V* = 0 and −0.3 V vs Ag/AgCl (maps of the pyrex/graphene sample can be found in the SI). Clear differences in ω_G_ are observed for the two potentials, as attested by the histograms in Figure 2a. Figure 2b depicts the average spectra of pyrex/graphene and ITO/graphene electrodes as a function of potential, where the voltage-dependence of the ω_G_ is evidenced. For a more complete analysis, we plot (Figure 2c) the full width at half-maximum of the G band (FWHM_G_) as a function of the potential for both systems. The FWHM of the Raman bands is a direct indicator of the phonon decay processes, and therefore, in the case of graphene, it is proportional to the electron–phonon coupling. In processed graphene samples, the electron–phonon coupling is affected by charge density variations resulting, among others, from the substrate self-doping effect. To assess that the strength of the electron–phonon coupling in graphene is analogous for pyrex and ITO substrates, so that we can correlate ω_G_ with the Fermi energy in both substrates, we first analyze FWHM_G_ as a function of the potential (Figure 2c). The FWHM of the Raman bands is a direct indicator of the phonon decay processes and, therefore, in the case of graphene, it is proportional to the electron–phonon coupling.^33^ The observed variations in FWHM_G_ are very similar in pyrex/graphene and ITO/graphene electrodes, confirming that the substrate conductivity does not affect the electron–phonon coupling in our systems. Once this is confirmed, we can evaluate the charge modulation by using the well-known correlation of the energy of the G phonon and the Fermi energy in graphene. Figure 2d presents the variation of graphene ω_G_ with applied voltage, showing the expected V shape as a function of potential. Interestingly, we observe an identical voltage dependence for graphene on pyrex and ITO. Analogous measurements performed on Au/graphene and Si/SiO_2_/graphene electrodes confirm these observations (see SI). Hence, the Raman spectroelectrochemistry results indicate that regardless of the substrate conductivity the same charge modulation can be effectively induced in graphene by applying an external potential.

**Figure 2:**
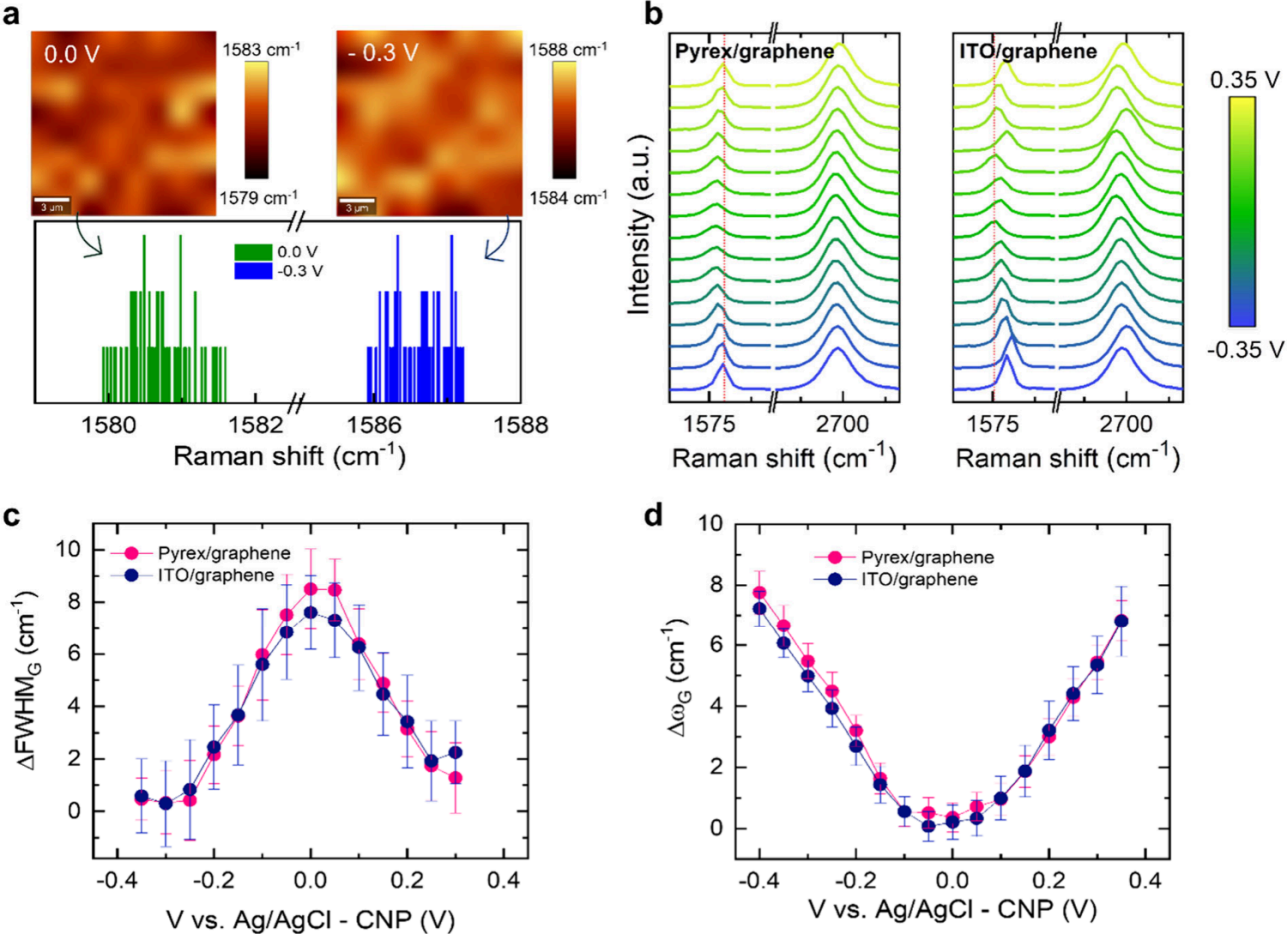
(a) Raman maps and corresponding histograms of the G band frequency, ω_G_, of an ITO/graphene electrode at 0 and −0.3 V. (b) Raman spectra obtained from pyrex/graphene and ITO/graphene electrodes measured in electrolyte while applying different voltages, as indicated in the lateral scale. Each Raman map is of 256 μm^2^. The vertical dashed red line is the approximate value of ω_G_ at the most positive and most negative potential. (c, d) Voltage-dependence of FWHM_G_ and ω_G_, respectively, for graphene electrodes prepared on pyrex (pink) and ITO (blue). The displayed statistical error bars correspond to the dispersion obtained in each acquired Raman map.

Considering the outcome of the electrochemical Raman study, the impedance behavior shown by the ITO/graphene electrode (i.e., 1 order of magnitude higher capacitance and no V-shape dependence with applied voltage) indicates that the conductive substrate contributes to the electrode impedance, which implies the existence of an electrical contact between the substrate and the bulk electrolyte. This contact, as discussed in the following, is enabled by intercalated water between graphene and the substrate. To confirm the behavior observed by PEIS and Raman spectroelectrochemistry, we performed measurements with graphene electrodes on other conductive (Au) and nonconductive (Si/SiO_2_) substrates (see SI).

To further elaborate on this hypothesis, we show (Figure 3a–c) experimental proof of water intercalation in our devices. Specifically, Figure 3a presents Raman spectra of graphene before and after immersion in water. Figure 3b displays a histogram of the frequency of the 2D band (ω_2D_) before and after immersion. After 2 h, the graphene ω_2D_ down-shifts ∼3.5 cm^–1^. This ω_2D_ shift is indicative of substrate–graphene decoupling, and has been previously attributed to water intercalation.^34,35^ Furthermore, the increased dispersion observed in ω_2D_ after immersion in water is evidence of the nonuniform way that water intercalates; this phenomenon has been tentatively explained by a nonuniform penetration of water through graphene’s grain boundaries.^17^Figure 3c depicts the ω_2D_ evolution during the time the graphene electrode is immersed in an aqueous electrolyte. It shows the ω_2D_ down-shifts progressively during the first 90 min. Kelvin probe force microscopy (KPFM) has been used to provide further evidence of the water intercalation between graphene and the substrate, as previously demonstrated.^36^ Contact potential difference (CPD) maps (see Figure S13 in the SI) unveil the presence of graphene regions with different CPD, which we attribute to water islands between graphene and the substrate. The observed inhomogeneous presence of intercalated water is in good agreement with literature.^14,37^

**Figure 3:**
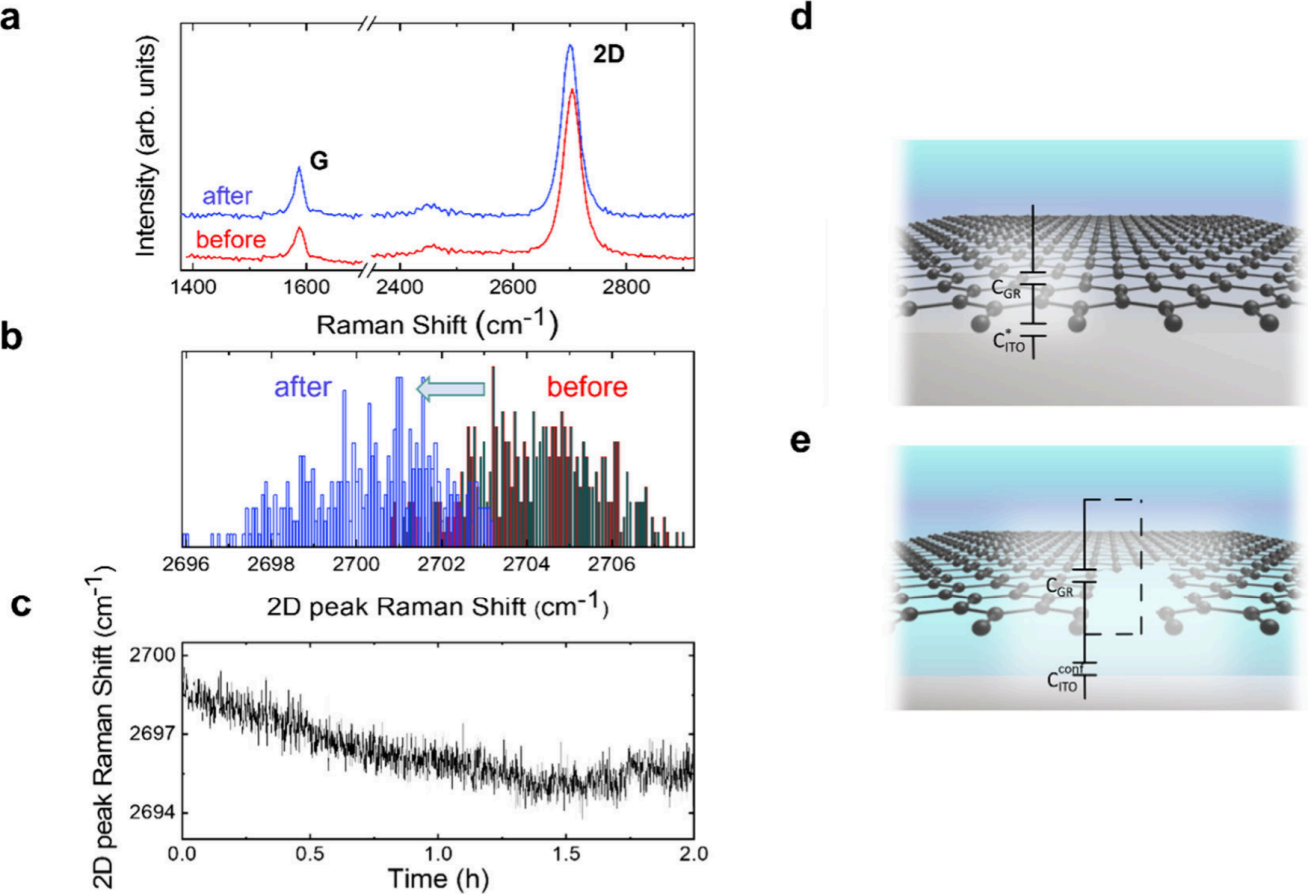
(a, b) Raman spectra of a graphene electrode and histogram of the ω_2D_ before (red) and after (blue) immersion in water. Results are from Raman maps of 400 μm^2^. (c) Evolution of ω_2D_ with time during electrode immersion in water. (d, e) Schematic illustrations representing the different coexisting interfaces formed on a graphene electrode in contact with an electrolyte for graphene areas in direct contact with the substrate and with intercalated water, respectively. *C*_Gr_ is the graphene capacitance, *C*_ITO_^*^ and *C*_ITO_^conf^ are the ITO capacitance contributions in the regions without and with intercalated water, respectively, which are explained in detail in the main text.

Once the existence of intercalated water is confirmed, its role in enabling the electrical contact between the conductive substrate and the electrolyte will be discussed. In the following, we describe the electrical circuit and the resulting capacitance of the different areas, without and with intercalated water, that coexist in the electrode (Figure 3d and e, respectively).

In the case of the graphene electrode area without water intercalation, the equivalent circuit considers the capacitive contributions of graphene (*C*_Gr_) and the substrate (*C*_ITO_^*^) connected in series:
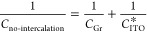
1

We consider *C*_Gr_ as our experimental capacitance obtained from the PEIS of pyrex/graphene (see Figure 1g). The ITO contribution, *C*_ITO_^*^, however, cannot be directly obtained from the PEIS measurements of bare ITO electrodes. In the case of the bare ITO electrodes, the measured capacitance, *C*_ITO_^bulk^, corresponds to the situation in which the ITO electrode is in direct contact with the bulk electrolyte, which is not the case for *C*_ITO_^*^. As explained in the Supporting Information, *C*_ITO_^*^ ≥ *C*_ITO_^bulk^. In this situation and considering that *C*_ITO_^bulk^ ≫ *C*_Gr_ (see Figure 1e), we can assume that *C*_no-intercalation_ will be governed by *C*_Gr_.

In the case of the graphene electrode areas with water intercalation, the presence of water enables an electrical connection between the substrate and the electrolyte; a more detailed discussion can be found in the SI. Considering this situation, the total capacitance in these regions, *C*_intercalation_, is governed by *C*_ITO_^conf^:
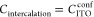
2where by *C*_ITO_^conf^ corresponds to the interfacial capacitance of ITO in direct contact with a nm-thin layer of confined water, and thus with dielectric properties different from bulk water.^38^ Since we do not have direct experimental access to *C*_ITO_^conf^, we performed numerical simulations considering reduced ion concentration and steric effects.

First, we modeled the capacitance of ITO in contact with bulk water, by *C*_ITO_^bulk^. To this end, we considered an ITO electrode in contact with bulk water but including a subnanometer insulating layer with reduced permittivity to account for the hydrophobicity properties of this material, showing good agreement with the experimental data (see Figure S15b). A similar approach is employed to obtain by *C*_ITO_^conf^ (Figure S15d), considering a layer of confined water with thickness (*t*_w_) and reduced permittivity (ε_w_) above the substrate (see SI for details). Figure 4b presents the calculated value by *C*_ITO_^conf^ for the case of one layer of confined water with varying ε_w_. The explored permittivity range accounts for the reduced dielectric properties of the confined water.^39^ The value calculated by *C*_ITO_^conf^ is significantly lower than that calculated by *C*_ITO_^bulk^ for all ε_w_; it shows a minimum near the charge neutrality point of the electrolyte (at around 0.2 V). In this voltage range, both the charge of the electrolyte and the confined water reach a minimum (see SI). With increasing ε_w_, by *C*_ITO_^conf^ increases and the above-mentioned minimum at 0.2 V becomes more evident. In contrast, a maximum at around −0.3 V is observed for lower ε_w_ values, resulting from a change in the contribution of the bulk and confined water regions to the capacitance. Around this voltage, the charge in the confined water region is high enough to partially screen the substrate effect, reducing the charge modulation in the bulk electrolyte (see SI). Figure 4c represents *C*_ITO_^conf^ calculated for one, two, and three layers of confined water, for a fixed permittivity (15.6ε_0_). A thicker confined water region leads to a reduction in the coupling between the bulk electrolyte and the ITO. Consequently, a higher impact of the confined water region is observed, which is translated into the maximum at around −0.3 V.

**Figure 4:**
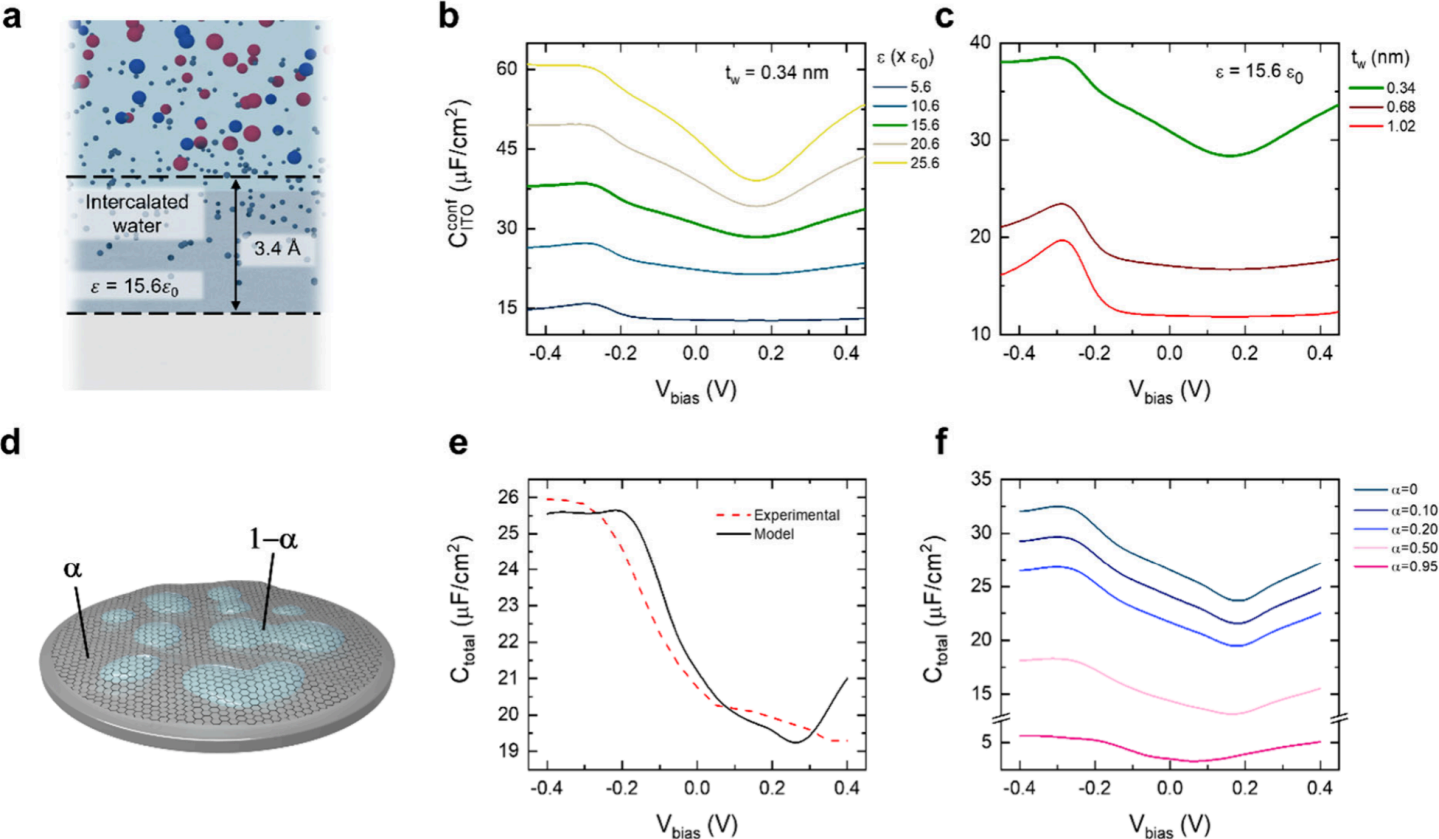
(a) Schematic illustration of the substrate–graphene–electrolyte interface. (b, c) Effect of the confined water permittivity and thickness, respectively, on the ITO capacitance (*C*_ITO_^conf^). (d) Schematic representation of a graphene electrode surface showing the different regions with (1 – α) and without (α) water intercalation. (e) Comparison of the experimental PEIS data, CPE of the ITO/graphene electrode, and simulated *C*_total_, with α = 0.23, ε_w_ = 13ε_0_ and *t*_w_ = 3.4 nm. (f) Simulated *C*_total_ for different values of α.

As experimentally found (Figure S13), water intercalation does not occur uniformly between graphene and the substrate, but in the form of clusters or islands.^37^ Therefore, to properly evaluate the total capacitance of the graphene electrode on ITO (*C*_total_), we took into account the contribution of two different types of regions in the electrode, with and without intercalated water (Figure 4d). We consider that these two regions are electrically connected in parallel, and therefore, *C*_total_ corresponds to the sum of the capacitance of each region, weighted by their surface coverage, α and 1 – α for areas without and with water intercalation, respectively:
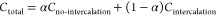
3

Our numerical model enables the simultaneous variation of ε_w_, *t*_w_, and α to reproduce the PEIS experimental data. Figure 4e shows the comparison of the experimental capacitance of the ITO/graphene electrode and the numerical simulation obtained considering α = 0.23. Our numerical model is able to replicate the experimental data and indicates that about 80% of the electrode area presents water intercalation, in good agreement with our KPFM results (Figure S13). Finally, we have assessed the sensitivity of the capacitance to the degree of water intercalation on the graphene electrodes. As previously commented, the degree of water intercalation may vary depending on the graphene quality (density of grain boundaries and defects) and the substrate hydrophobicity. Our model can predict the electrochemical response of graphene electrodes at limit situations of degree of water intercalation. Figure 4f depicts *C*_total_ for a range of α (0–0.95), revealing how increasing water intercalation enhances the graphene-substrate decoupling by means of the electrical direct connection of the substrate with the electrolyte, eventually leading to an electrode totally governed by the substrate–electrolyte interfacial capacitance in the case of conductive substrates.

## Conclusions

In this work, we have investigated how interfacial phenomena at the substrate-graphene-electrolyte govern the performance of graphene electronic devices. We found significant differences in the voltage dependence of the impedance of graphene electrodes supported on pyrex and on ITO substrates. However, Raman spectroelectrochemistry experiments demonstrate that such differences in impedance are not associated with differences in the efficacy of charge modulation of differently supported graphene. We found that it was possible to equally modulate graphene’s surface charge, independently from the conductivity of the substrate. To reconcile both observations, i.e., identical charge modulation efficacy but very different interfacial capacitances for graphene electrodes on conductive and insulating substrates, we describe how the intercalation of water between graphene and the substrate allows a direct electrical path between the substrate and the bulk electrolyte. Our results reveal the coexistence of two different domains in the graphene electrodes, one in which graphene is in direct contact with the substrate and one in which water is intercalated between graphene and the substrate. We conducted numerical simulations to rationalize the impact of the intercalated water layer on the interfacial capacitance by varying the permittivity and thickness of this confined water, as well as the total surface that confined water occupies in the electrode. Our findings contribute to the understanding of the impact of water intercalation on the electrical response of graphene-based devices and offer valuable insights into the methods used to measure and model this phenomenon.

## Supporting Information Available

Materials and Methods: graphene CVD growth and wet transfer, graphene macroelectrodes preparation, PEIS measurements and fitting, Raman spectroscopy and spectroelectrochemistry, AFM/KPFM, numerical simulations. Figure S1: Schematic top view of the electrodes design. Figure S2: Cyclic voltammetry of pyrex/graphene. Figure S3: Pyrex/graphene and ITO/graphene Bode curves with complete data set and fitting. Figure S4: Bare ITO Bode curves with complete data set and fitting. Figure S5: Statistics on CPE modulation with voltage of the pyrex/graphene and ITO/graphene electrodes. Figure S6: Bare ITO UPS measurement and work function calculation. Figure S7: Bare ITO carrier density calculation. Figure S8: Au/graphene and bare Au Bode curves with complete data set and fitting. Figure S9: Raman spectroelectrochemistry on an Au/graphene electrode. Figure S10: Si/SiO_2_/graphene Bode curves with complete data set and fitting. Figure S11: Raman spectroelectrochemistry on an Si/SiO_2_/graphene electrode. Figure S12: ω_G_ Raman maps of a pyrex/graphene electrode. Figure S13: KPFM on a graphene device before and after PBS immersion. Figure S14: Equivalent electronic circuit describing electrode areas with water intercalation. Figure S15: Simulation of *C*_ITO_^bulk^ and *C*_ITO_^conf^. Figure S16: Charge density profiles of the confined water region and bulk electrolyte as a function of the bias for different values of permittivity of the intercalated water. Figure S17: Contribution of the confined water region and the bulk electrolyte to the total capacitance for different values of permittivity of the intercalated water. Figure S18: Charge density profiles of the confined water region and bulk electrolyte as a function of applied voltage for different thicknesses of the intercalated water. Figure S19: Contribution of the confined water region and the bulk electrolyte to the total capacitance for different thicknesses of the intercalated water (PDF)

## Abbreviations

PEIS: potentiostatic electrochemical impedance spectroscopy
RE: reference electrode
CE: counter electrode
CPE: constant phase element
CNP: charge neutrality point
EDL: electrochemical double layer
DC: direct current
FWHM: full width half-maximum
AFM: atomic force microscopy
KPFM: Kelvin probe force microscopy
CPD: contact potential difference
CVD: chemical vapor deposition
SEM: scanning electron microscopy
PBS: phosphate buffer saline
PDMS: polydimethylsiloxane
PMMA: poly(methyl methacrylate)
RIE: reactive ion etching
UPS: ultraviolet photoelectron spectroscopy
IP: ionization potential
WE: working electrode
