# Supplementary material for: Interfacial Phenomena Governing Performance of Graphene Electrodes in Aqueous Electrolyte
Source: Nano Lett. 2024 Sep 4;24(37):11376–84. doi: 10.1021/acs.nanolett.4c01808 (PMC11421073; doi:10.1021/acs.nanolett.4c01808)
Supplement: Supplementary file 1 — nl4c01808_si_001.pdf [file nl4c01808_si_001.pdf]

Interfacial phenomena governing performance of graphene electrodes in aqueous electrolyte

Marta Delgà-Fernández[1], Alejandro Toral-Lopez[2], Anton Guimerà-Brunet[3,4], A. Pablo Pérez-Marín[1], Enrique G. Marin[2], Andrés Godoy[2], Jose A. Garrido[1,5], Elena del Corro[1]

[1]Catalan Institute of Nanoscience and Nanotechnology (ICN2), CSIC and BIST, 08193 Bellaterra, Spain
[2]Pervasive Electronics Advanced Research Laboratory (PEARL), Department of Electronics and Computer Technology, University of Granada, 18071 Granada, Spain
[3]Institut de Microelectrònica de Barcelona (IMB-CNM), CSIC, Esfera UAB, 08193 Bellaterra, Spain
[4]Centro de Investigación Biomédica en Red en Bioingeniería, Biomateriales y Nanomedicina (CIBER-BBN), 28029 Madrid, Spain
[5]ICREA, 08010 Barcelona, Spain

Corresponding author's email address: elena.delcorro@icn2.cat

KEYWORDS: graphene, interfacial phenomena, electrical double layer, water intercalation

Supporting Information

Materials and Methods: graphene CVD growth and wet transfer, graphene macroelectrodes preparation, PEIS measurements and fitting, Raman spectroscopy and spectroelectrochemistry, AFM/KPFM, numerical simulations.

Figure S1: Schematic top view of the electrodes design.

Figure S2: Cyclic voltammetry of pyrex/graphene.

Figure S3: Pyrex/graphene and ITO/graphene Bode curves with complete data set and fitting.

Figure S4: Bare ITO Bode curves with complete data set and fitting.

Figure S5: Statistics on CPE modulation with voltage of the pyrex/graphene and ITO/graphene electrodes.

Figure S6: Bare ITO UPS measurement and work function calculation.

Figure S7: Bare ITO carrier density calculation.

Figure S8: Au/graphene and bare Au Bode curves with complete data set and fitting.

Figure S9: Raman spectroelectrochemistry on an Au/graphene electrode.

Figure S10: Si/SiO_2/graphene Bode curves with complete data set and fitting.

Figure S11: Raman spectroelectrochemistry on an Si/SiO_2/graphene electrode.

Figure S12: ω_G Raman maps of a pyrex/graphene electrode.

Figure S13: KPFM on a graphene device before and after PBS immersion.

Figure S14: Equivalent electronic circuit describing electrode areas with water intercalation.

Figure S15: Simulation of C_{ITO}^{bulk} and C_{ITO}^{conf}.

Figure S16: Charge density profiles of the confined water region and bulk electrolyte as a function of the bias for different values of permittivity of the intercalated water.

Figure S17: Contribution of the confined water region and the bulk electrolyte to the total capacitance for different values of permittivity of the intercalated water.

Figure S18: Charge density profiles of the confined water region and bulk electrolyte as a function of applied voltage for different thicknesses of the intercalated water.

Figure S19: Contribution of the confined water region and the bulk electrolyte to the total capacitance for different thicknesses of the intercalated water.

MATERIALS AND METHODS

Graphene CVD growth and wet transfer: Graphene was grown on a 4.5×8 cm copper foil (Graphene Platform copper foil 99.95%, thickness: 0.035 mm) via CVD. Before graphene growth, the copper foil was electropolished for 5 min with a fixed current density of 62 mA/cm^2 in a solution of H_2O, 0.5 L of H_3PO_4, 0.5 L of ethanol, 0.1 L of isopropanol and 10 g of urea. For the CVD process, the Cu foil was loaded into a planar quartz tube heated by a three-zone oven. A thermal annealing was performed at 1050°C for 90 minutes under an Ar atmosphere at ~100 mbar. The growth step consisted of increasing the temperature to 1065°C and introducing H_2 and CH_4 flows during 20 minutes at ~25 mbar. The sample was finally quenched down to room temperature by placing the tube out of the heating zone. For the graphene film transfer from the Cu to the desired substrate, poly(methylmethacrylate) (PMMA A4) was spun onto the Cu/graphene and dried at room temperature for 12 h. Then, graphene backside was removed by RIE (40W HF, 80 mTorr, Ar:O_2 40:40 sccm, 6 min). Afterwards, the sample was left floating on a $FeCl_3$/HCl solution for over 12 h and cleaned two times with deionized water (2+1 h),

before transferring it onto the final substrate, which is previously rinsed with isopropanol and deionized water. Subsequently, the sample was dried at 40°C in a hotplate for 2 h and under P~10-7 mbar, up to 180°C, 1 min in a heating ramp of 6°C/min. Finally, the PMMA was dissolved in acetone and isopropanol (30 min each). The quality of the graphene layer was checked by Raman spectroscopy and scanning electron microscopy (SEM).

Graphene macroelectrodes: 5×5 mm graphene pieces were transferred to the desired substrates. For pyrex/graphene electrodes, Ti/Au (15/150 nm) contacts were evaporated by electron-beam evaporation on a 1.5x1.5 cm pyrex substrate, leaving a 2 mm diameter circle in the centre, which was subsequently covered with the 5×5 mm graphene sheet. For the ITO/graphene electrodes, Ti/Au contacts were evaporated leaving a 10 mm diameter circle in the centre, where the graphene sheet was placed without being in direct contact with the Ti/Au. The passivation was made with PDMS.

Potentiostatic Electrochemical Impedance spectroscopy measurements and fitting: PEIS measurements were performed using a potentiostat (BioLogic SP-200) in a three-electrode configuration. All solutions were prepared with phosphate-buffered saline (PBS 150 mM, purchased from Merck). Custom python scripts have been used for fitting the measured PEIS data into the equivalent circuit parameters described below by means of the Levenberg–Marquardt method.

Raman spectroscopy and spectroelectrochemistry: The Raman maps were acquired in a WITec spectrometer in backscattering configuration, with a 600 gr/nm grating 488 nm excitation laser (2 mW power) focused with a 63x water objective on the sample, immersed in the electrolyte. Raman spectroelectrochemistry was performed in a two-electrode configuration: WE (graphene electrode) and RE (Ag/AgCl) using the same potentiostat as for the PEIS measurements. For dry measurements, a 50x objective was used. A spatial resolution below 1 μm was obtained. The spectral resolution was of 3 cm^{-1}/pixel. Raman maps were fitted to 2 Lorentzian peaks corresponding to the G and 2D graphene bands, after performing a background subtraction.

AFM/KPFM: For Kelvin Probe Force Microscopy (KPFM), we used an MFP-3D Origin AFM (Asylum Research by Oxford Instruments) and a Pt/Ir coated probe (Nanosensors, PPP-NCHPt-20). The KPFM measurement was performed during constant-height reverse scans after AFM topography imaging, and the tip was lifted 10 nm above the sample surface after the topographic scan, to perform the electrical scan. The tip-samples contact potential difference

was acquired by applying a DC feedback voltage (V_{DC}) to cancel the component of the electrostatic force between the tip and the sample, while the tip oscillated at frequency (f) proportional to the gradient of the tip-sample capacitance along the surface normal, $F_f \propto (\frac{\partial C}{\partial z})(V_{CPD} - V_{DC})$.

Numerical simulations: The simulations were carried out with an in-house code for the self-consistent numerical solution of the Poisson equation along with the charge in the different regions of the structure. The charge in the electrolyte is evaluated using the Modified-Boltzmann equation along with the chemical reactions involving the PBS.

Schematic top view of the electrodes design

To prepare the electrodes, first, gold contacts were evaporated by electron beam leaving a circular area at the centre of each substrate: 3 mm diameter for the pyrex and 10 mm diameter for the ITO electrodes. Then, 25 mm^2 graphene sheets (5×5 mm), grown by CVD on copper foils, were transferred using the wet transfer method (see Methods above). Next, samples were manually passivated using polydimethylsiloxane (PDMS), resulting in 2 mm diameter pyrex/graphene and ITO/graphene electrodes.

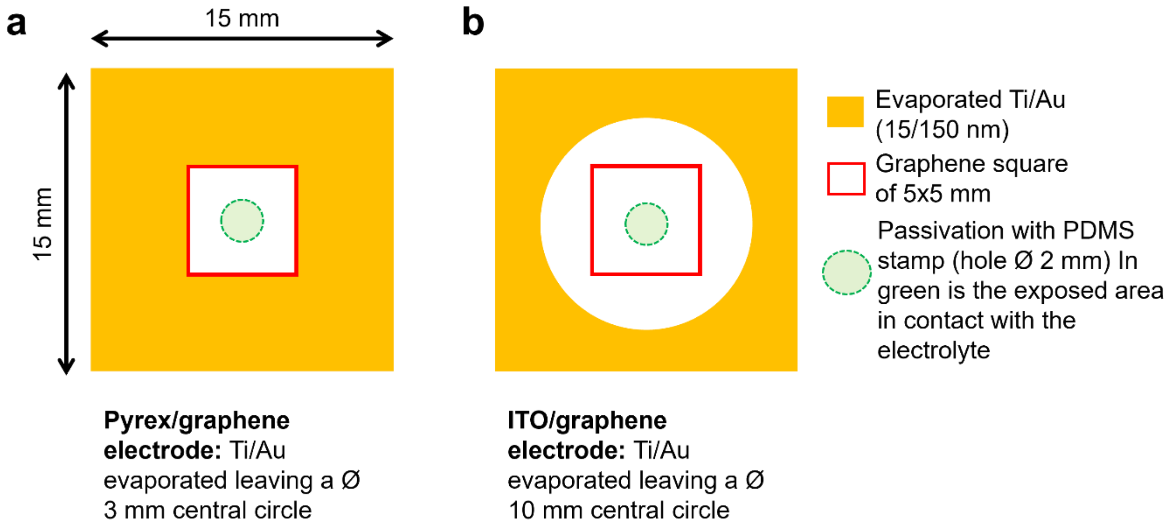

Figure S1: Schematics of the electrodes prepared for the study. Details about graphene growth and transfer Ti/Au evaporation and electrodes passivation are described in Methods.

Cyclic voltammetry of a graphene electrode

The PEIS measurements presented in this work are performed in a voltage range between -0.35 V and 0.35 V, accounting for the potential window of graphene. This is confirmed by the cyclic voltammetry measurement shown in Figure S2.

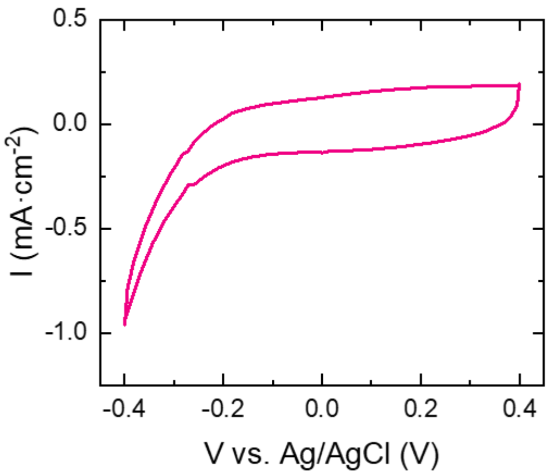

Figure S2: Cyclic voltammetry measured on a graphene/pyrex electrode in PBS, using the 3-electrode experimental setup represented in Figure 1a of the main text.

Pyrex/graphene and ITO/graphene Bode curves with complete data set and fitting

The total impedance $Z(\omega)$ of the electrode is calculated by $Z(\omega) = \frac{-R_{sh} J_0(bL,\omega)}{2\pi L b J_1(bL,\omega)}$, where L is the electrode radius, J_0 and J_1 the hyperbolic Bessel functions of the first kind and of the zeroth and first order respectively, and $b^2 = -\frac{R_{sh}}{2\pi L \frac{1}{CPE(j\omega)^a}}$. A more detailed description of this expression is referenced[1].

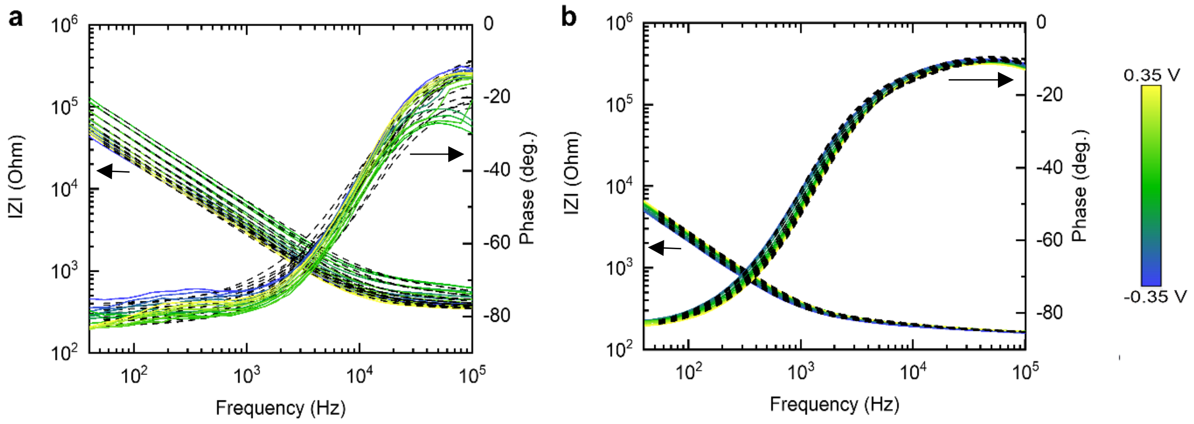

c

Distributed model equivalent circuit					
	**CPE**	**a**	$\mathbf{R_n}$	$\mathbf{R_{ct}}$	$\mathbf{R_c}$
**(a) Pyrex/graphene**	$1\cdot10^{-6} < \mathbf{3\cdot10^{-6}} < 1$	0.85 < 0.9 < 1	$500 < \mathbf{5\cdot10^{3}} < 8\cdot10^{3}$	$10\cdot10^{6} < \mathbf{50\cdot10^{6}} < 100\cdot10^{6}$	335 < **400** < 630
**(b) ITO/graphene**	$1\cdot10^{-6} < \mathbf{3\cdot10^{-6}} < 1$	0.9 < **0.95** < 1	$1^{3} < \mathbf{1.1\cdot10^{4}} < 1.5\cdot10^{4}$	$1^{6} < \mathbf{5^{6}} < 1^{8}$	150 < **150** <154

Figure S3: Complete data set of Bode curves for (a) pyrex/graphene and (b) ITO/graphene. Each colour of the continuous lines corresponds to each applied voltage, as indicated by the legend. The fitting of the experimental data is showed by the black dashed lines in the graphs. (c) Table showing the fitting boundaries for each parameter of the equivalent circuit used to determine the CPE (electrode/electrolyte capacitance) from PEIS data. R_n and R_{ct} (accounting for the processes at the electrode involving charge transfer), are the two contributions of R_{sh}.

Bare ITO Bode curves with complete data set and fitting

a

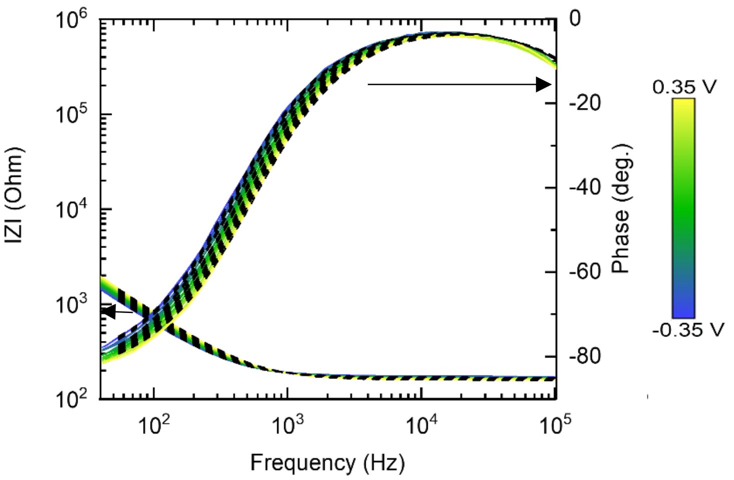

b

Simple model equivalent circuit				
	CPE	a	R_{sh}	R_s
**Bare ITO**	$8 \cdot 10^{-8}$ < $\mathbf{50 \cdot 10^{-8}}$ < $300 \cdot 10^{-7}$	0.9 < **0.95** < 1	$0.2 \cdot 10^5$ < $\mathbf{0.5 \cdot 10^5}$ < $10 \cdot 10^6$	145 < **155** < 170

c

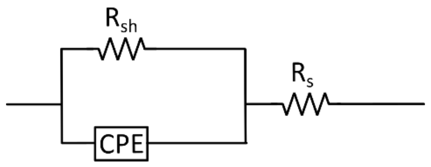

Figure S4: (a) Complete data set of Bode curves including fittings (black dashed lines) for bare ITO. (b) Table indicating the fitting boundaries for each parameter of the equivalent circuit, showed in (c), used in this case to calculate the total impedance of the electrode.

Statistics on CPE modulation with voltage of the pyrex/graphene and ITO/graphene electrodes

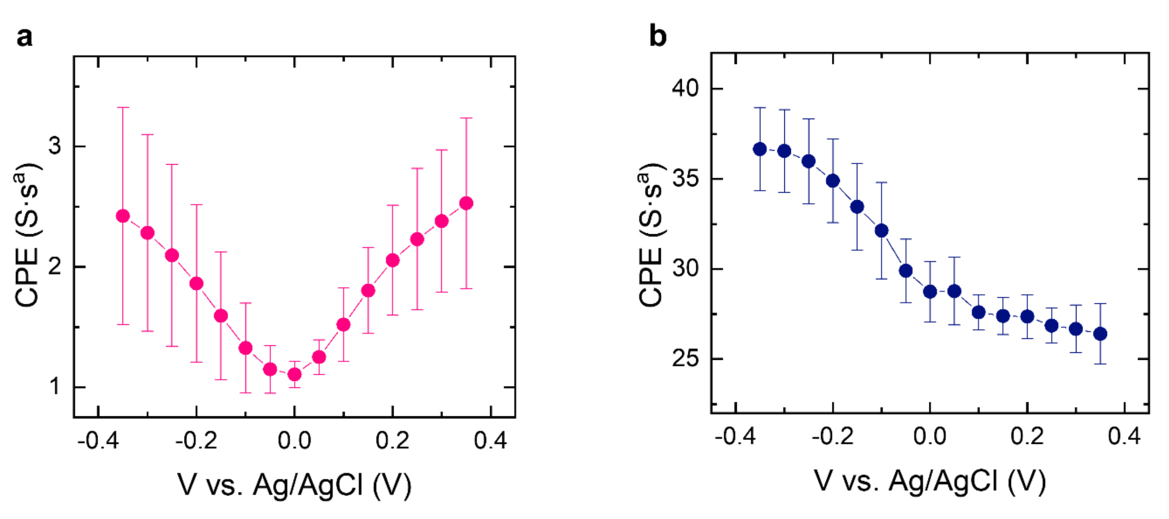

Figure S5: Statistical CPE modulation with voltage of (a) pyrex/graphene and (b) ITO/graphene electrodes. n = 3 samples.

Bare ITO UPS measurement and work function calculation

The work function is calculated first obtaining the ionization potential (IP) according to the procedure proposed by Diaz et al[2].: $IP = 21.2 - E_{cut-off} - E_{VB}^{F}$ and then the work function is determined by $\phi = IP - E_{VB}^{F}$.

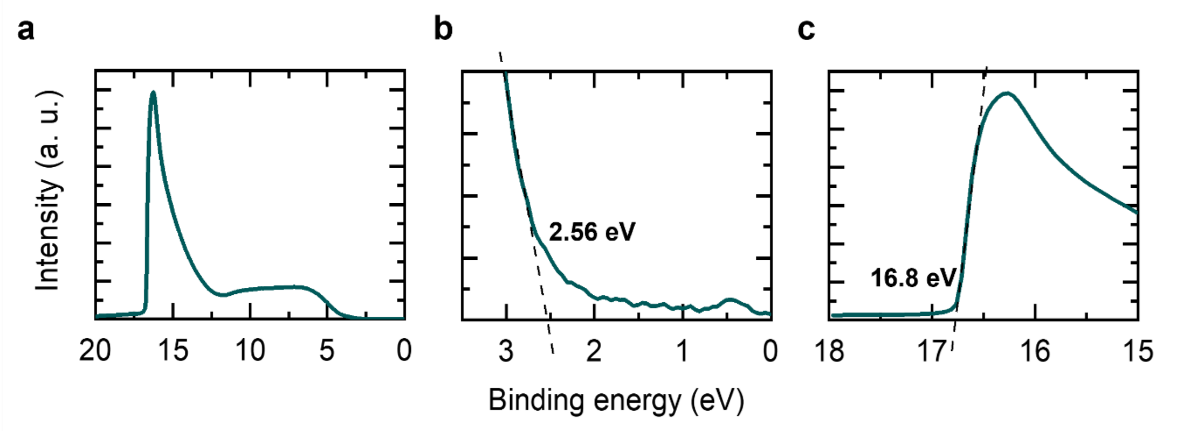

Figure S6: (a) UPS spectra of bare ITO. Zoom in the (b) valence band and (c) UPS secondary electron edge.

Bare ITO carrier density calculation

The carrier density of bare ITO ($1.12 \cdot 10^{18}$ cm^{-3}) is calculated using the slope of the Mott-Schottky plot depicted in Figure S6 ($8.42 \cdot 10^{-2}$ $\mu F^2/V$) and considering the Mott-Schottky equation: $\frac{1}{C^2} = (\frac{2}{e\varepsilon\varepsilon_0\, A^2 N_D})(E - E_{FB} - \frac{\kappa T}{e})$ for n-type semiconductors.

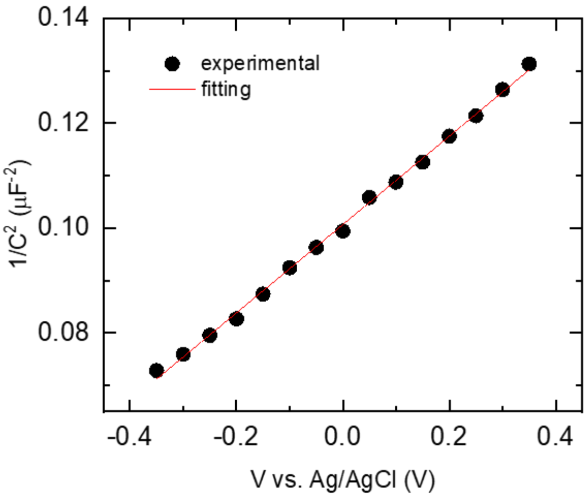

Figure S7: Mott-Schottky plot for bare ITO. C values are obtained from the PEIS measurements.

Au/graphene and bare Au electrodes

Figure S8 shows the PEIS study on Au/graphene and bare Au electrodes. This includes the Bode curves, the CPE-V modulation and the fitting boundaries used in the analysis. Figure S9 shows Raman spectroelectrochemistry measurements on Au/graphene electrodes. Charge modulation is observed in Raman measurements, regardless of the lack of CPE-V modulation measured by PEIS. These results confirm that what is observed in the ITO/graphene system, when graphene is supported on a conductive substrate, like metallic Au, water intercalation phenomenon occurs, leading to the additional contribution in series of Au capacitance to the PEIS.

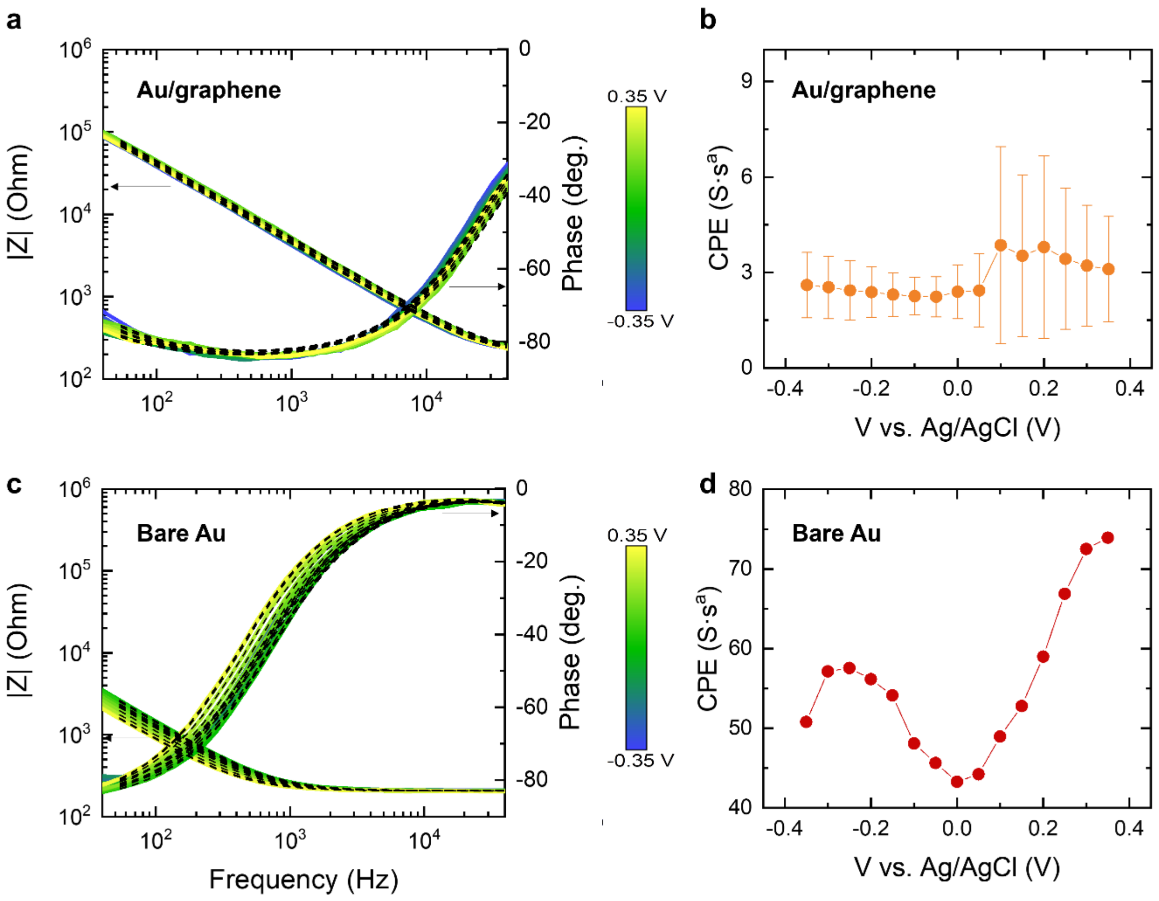

e

Simple model equivalent circuit				
	**CPE**	**a**	**R_{sh}**	**R_s**
**Au/graphene**	$1\cdot10^{-8}$ < $\mathbf{5\cdot10^{-8}}$ < $200\cdot10^{-7}$	0.86 < 0.9 < 0.99	$0.4\cdot10^{6}$ < $\mathbf{1\cdot10^{6}}$ < $10\cdot10^{6}$	185 < **200** < 210
**Bare Au**	$1\cdot10^{-8}$ < $\mathbf{5\cdot10^{-8}}$ < $200\cdot10^{-7}$	0.85 < **0.9** < 0.99	$0.4\cdot10^{6}$ < $\mathbf{1\cdot10^{6}}$ < $10\cdot10^{6}$	185 < **200** < 215

Figure S8: (a, c) Bode curves for Au/graphene and bare Au electrodes. The fitting of the experimental data is showed by the black dashed lines. Each colour of the continuous lines corresponds to each applied voltage, as indicated by the legend. (b, d) CPE modulation with voltage of Au/graphene and bare Au samples. (e) Table showing the fitting boundaries for each parameter of the simple model equivalent circuit used (see Figure S3c) to determine the CPE (electrode/electrolyte capacitance) from PEIS data.

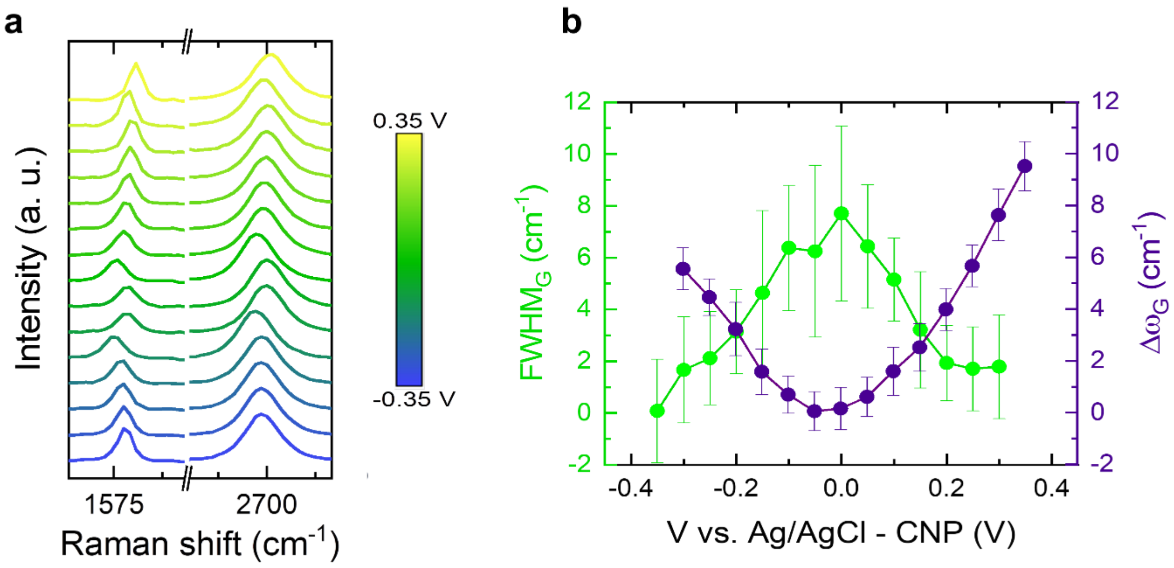

Figure S9: (a) Raman spectra obtained from an Au/graphene electrode measured in electrolyte while applying different voltages, as indicated in the lateral scale. Each Raman maps measured are of 256 μm^2. (b) Voltage-dependence of the full-width-half-maximum of the G band ($FWHM_G$) and ω_G for graphene electrodes prepared on Au. The displayed statistical error bars correspond to the dispersion obtained in each acquired Raman map.

Si/SiO_2/graphene electrodes

Figure S10 shows the PEIS study on Si/SiO_2/graphene electrodes. This includes the Bode curves, the CPE-V modulation and the fitting boundaries used in the analysis. Figure S11 shows Raman spectroelectrochemistry measurements on Si/SiO_2/graphene electrodes. Analogously to the pyrex/graphene case, the CPE shows a V-shape behaviour with the applied voltage, and the charge modulation of graphene is confirmed by Raman. The strength of the electron-phonon coupling depends on the graphene-substrate binding strength[3]. Note that in our experiments of graphene supported on silicon oxide we observe a slightly different electron-phonon coupling than for the other three systems under study (pyrex, ITO and Au). Consequently, in the case of graphene on silicon, the modulation of the Fermi level derived from Raman is not directly comparable.

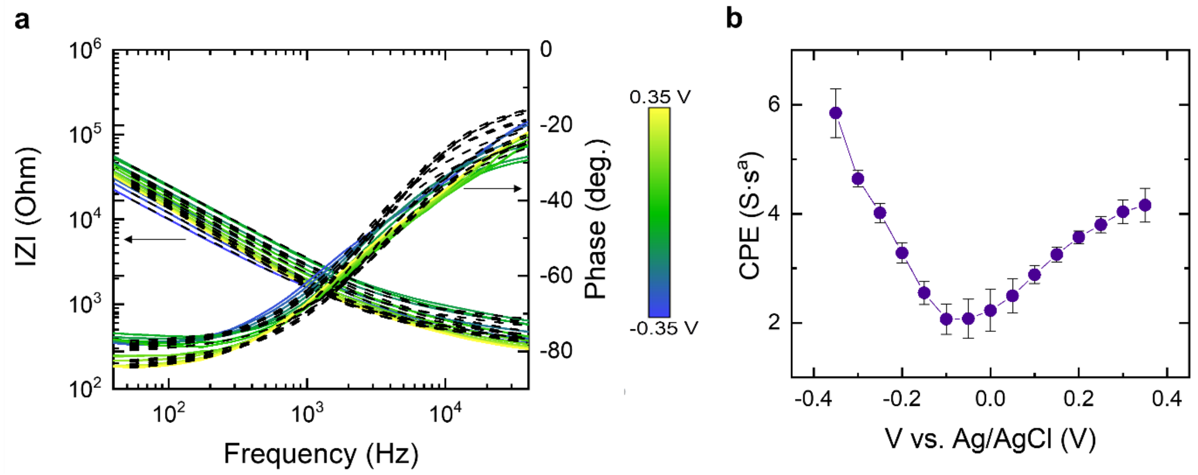

c

Distributed model equivalent circuit					
	**CPE**	**a**	**R_n**	**R_{ct}**	**R_c**
**Si/SiO_2/graphene**	$1 \cdot 10^{-6}$ < $\mathbf{3 \cdot 10^{-6}}$ < 1	0.8 < 0.9 < 1	500 < $\mathbf{5 \cdot 10^3}$ < $8 \cdot 10^3$	$10 \cdot 10^6$ < $\mathbf{50 \cdot 10^6}$ < $100 \cdot 10^6$	200 < **250** < 800

Figure S10: (a) Bode curves for Si/SiO_2/graphene. The fitting of the experimental data is showed by the black dashed lines. Each colour of the continuous lines corresponds to each applied voltage, as indicated by the legend. (b) CPE modulation with voltage of Si/SiO_2/graphene electrodes. n = 3 samples. (c) Table showing the fitting boundaries for each parameter of the distributed model equivalent circuit used to determine the CPE (electrode/electrolyte capacitance) from PEIS data.

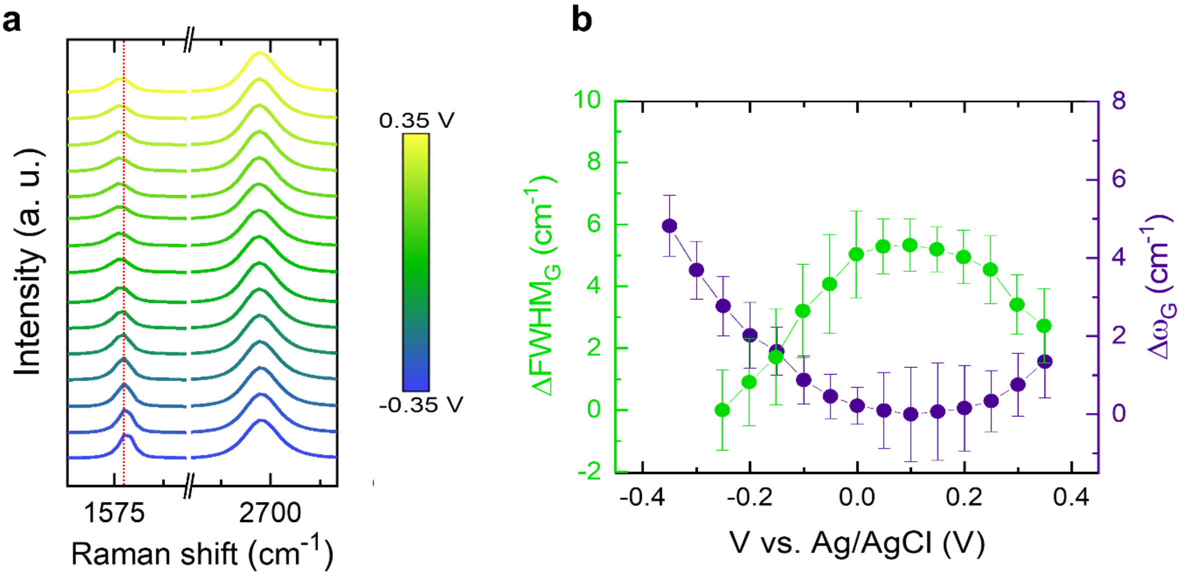

Figure S11: (a) Raman spectra obtained from a Si/SiO_2/graphene electrode measured in electrolyte while applying different voltages, as indicated in the lateral scale. Each Raman maps measured are of 256 μm^2. (b) Voltage-dependence of the full-width-half-maximum of the G band ($FWHM_G$) and ω_G for graphene electrodes prepared on Si/SiO_2. The displayed statistical error bars correspond to the dispersion obtained in each acquired Raman map.

ω_G Raman maps of a pyrex/graphene electrode

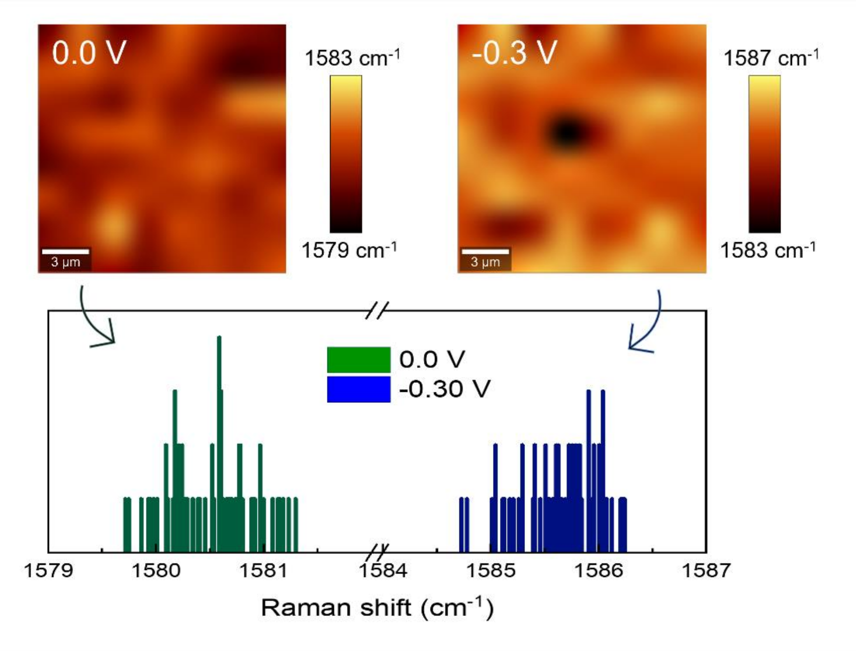

Figure S12: Raman maps and histograms of the ω_G of a pyrex/graphene electrode at 0 V and -0.30 V.

KPFM on a graphene device before and after PBS immersion

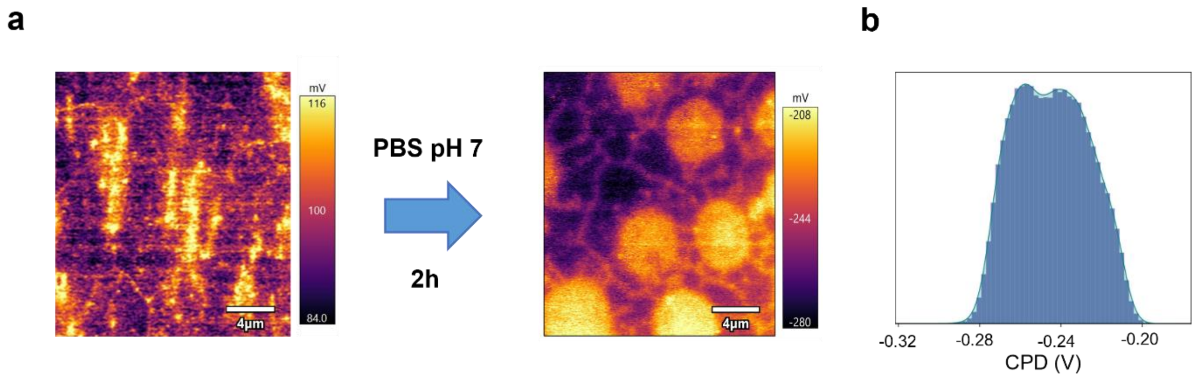

Figure S13: (a) CPD map obtained by KPFM on a 120 × 30 µm graphene device (SiO_2/Si substrate) before and after immersion in PBS for 2 hours and (b) corresponding CPD values histogram distribution.

Considerations on the analysis of $\boldsymbol{C_{no-intercalation}}$

Regarding the regions without intercalated water ($\boldsymbol{C_{no-intercalation}}$), we assume that $C_{no-intercalation} \cong C_{Gr}$, where C_{Gr} is the capacitance measured from PEIS studies on the pyrex/graphene structure. C_{ITO}^{*} can be (roughly) described by a two-parallel plate element, in correspondence with the beginning of the depletion region of the ITO and its surface, being the distance between them the size of the depletion region. C_{ITO}^{bulk}, on the other hand, is spatially defined by the electrolyte (the first plate, ultimately determined by the closest approach of ions to the surface) and the substrate (the second plate, associated to the depletion region in the ITO layer). Taking into account these descriptions of both capacitances, it can be inferred that C_{ITO}^{*} $> C_{ITO}^{bulk}$. Considering that $C_{Gr} << C_{ITO}^{bulk}$, as extracted from the experimental results, and that $C_{ITO}^{*} \geq C_{ITO}^{bulk}$, then we can conclude that $C_{Gr} << C_{ITO}^{*}$. Introducing this into Eq. (2) of the main text, we achieve $C_{no-intercalation} \cong C_{Gr}$.

Equivalent electronic circuit describing electrode areas with water intercalation

Regarding $C_{intercalation}$, Figure S14 schematizes the complete capacitance model for these regions. Here, a capacitance corresponding to the graphene/confined water, C_{Gr}^{conf}, is short-circuited with C_{ITO}^{conf}, and thus not contributing to $C_{intercalation}$.

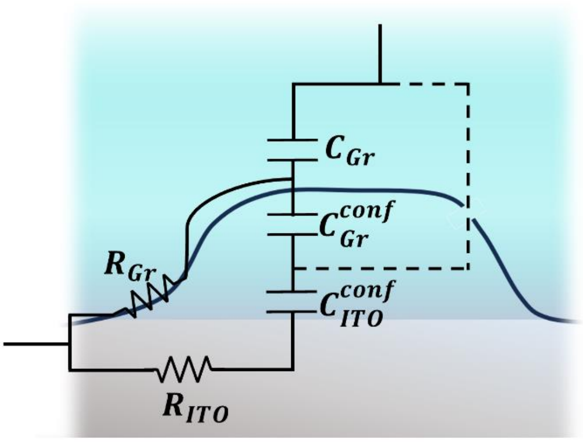

Figure S14: Schematics of the electronic circuit representing the areas of the ITO/graphene electrodes with water intercalation.

NUMERICAL SIMULATIONS

General considerations

All the simulations of this work were carried out with an *in-house* code that deals with the self-consistent solution of the Poisson equation, that relates the electrostatic potential V and charge density distribution ρ in the structure:

$$\nabla(\varepsilon\nabla V) = \rho \tag{S.1}$$

The charge density in the electrolyte is determined by the ions in the solution. The relation between the electrostatic potential and the ion distribution is defined through the modified Boltzmann equation:

$$c_i = c_{0,i}\frac{e^{-z_i(V-V_{ref})/k_BT}}{1-2\frac{c_{0,i}}{c_{max,i}}\left(1-\cosh\left(q|z_i|\frac{V-V_{ref}}{k_BT}\right)\right)} \tag{S.2}$$

where V_{ref} is the reference potential, $c_{0,i}$ the bulk concentration of *i-th* ion, z_i its valence and $c_{max,i}$ its maximum concentration. This latter is the key element to add steric effects in the simulation. More specifically, these are included through the hard sphere model, where each ion is characterized by an effective ionic radius R_{eff} that is related with the maximum allowed concentration $\left(c_{max} = 1/R_{eff}\right)$. More details of this model can be found in cited references[4,5].

Modelling of C_{ITO}^{bulk}

The bare ITO substrate (Figure S15a) is modelled as a boundary condition, which agrees with its conductive nature. On top of it, an insulating layer is considered to model the hydrophobicity of this material. These parameters are set according to the typical profiles obtained with Molecular Dynamic calculations near hydrophobic and hydrophilic surfaces[6] (Figure S15a right). The thickness of the layer is set to 1Å, which is the approximate size of the plateau region for the hydrophobic profile in Figure S15a right. The dielectric constant of this region is tuned to fit the experimental data, which is achieved for $18\varepsilon_0$, as shown in Figure S10b.

Modelling of C_{ITO}^{conf}

For the region with confined water at the ITO/graphene electrodes, we considered a structure like the previous one for the bare ITO/bulk electrolyte scenario. In this case, however, the insulating layer is substituted by a 3.4Å region with a reduced dielectric constant $(13\varepsilon_0)$ where

only H^+ and OH^- ions are allowed to enter. Steric effects are included in this region considering an effective radius of 1.5 nm (Figure S15c).

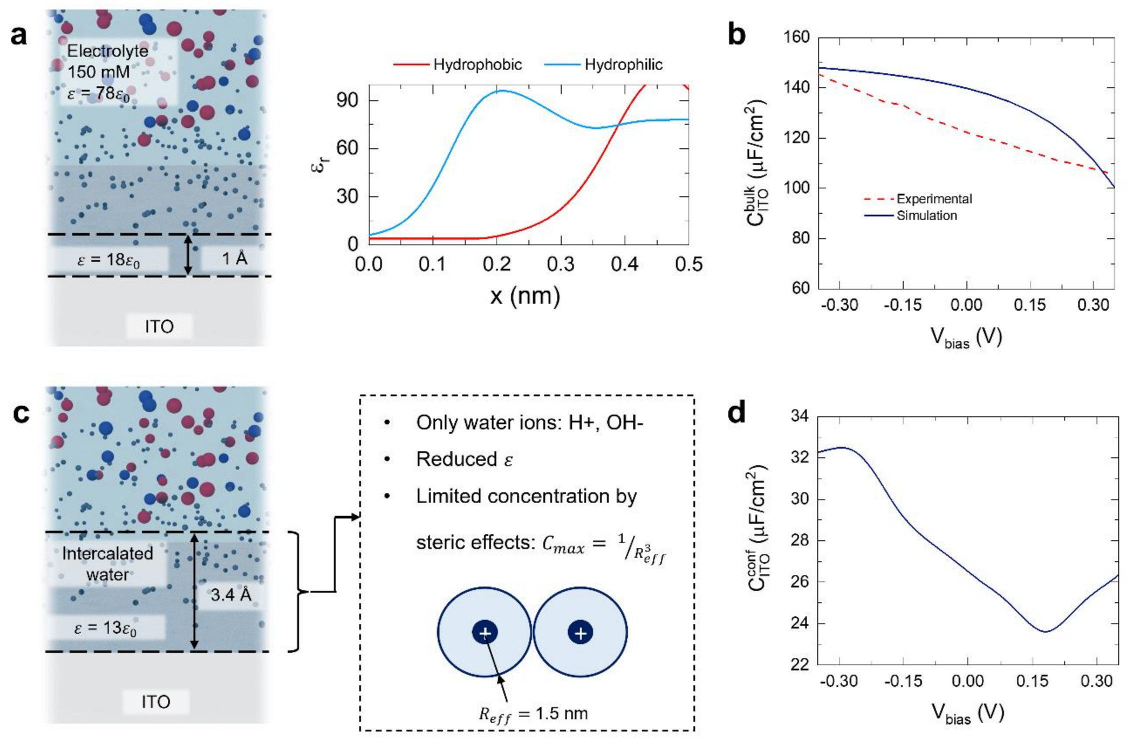

FIGURE S15: (a) Simulation of the bare ITO-bulk electrolyte system. (b) Modelled C_{ITO}^{bulk} compared with the experimental data obtained by PEIS. (c) Parameters considered for the simulation of the ITO/confined water/bulk electrolyte structure. (d) Modelled C_{ITO}^{conf}.

Impact of ε_w and t_w: extended analysis

Additional simulations were carried out for the structure depicted in Figure S15c focused on sweeping the dielectric constant ε_w and the thickness t_w of the confined water region. First parameter swept is the permittivity. Figure S16 depicts the charge density profile for the confined water region (N_w) and bulk electrolyte (N_B), while Figure S17 shows the contribution of each of these regions to the total capacitance.

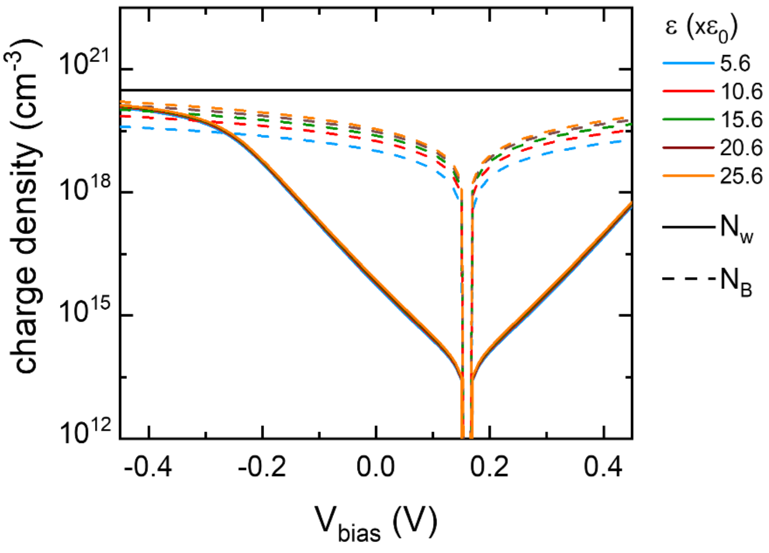

Figure S16: Charge density profiles of the confined water region (N_w) and bulk electrolyte (N_B) as a function of the bias for different values of ε_w. Black line indicates the limit defined by steric effects.

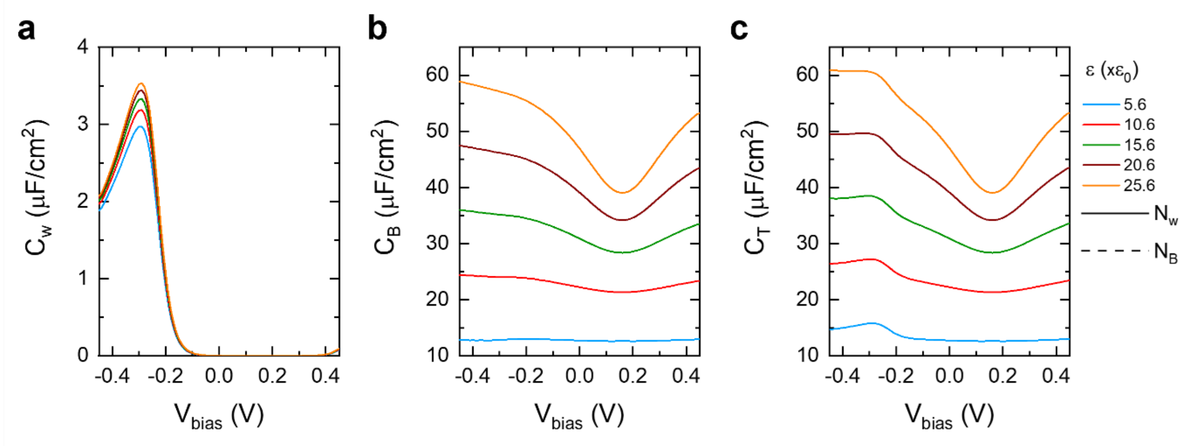

Figure S17: (a,b) Capacitances between the ITO substrate and each of the regions of the structure and (c) total capacitance of the structure.

The charge density profiles in Figure S16 show that confined water barely change its behaviour, while the bulk electrolyte is the region more impacted by ε_w. Regarding N_w, we can observe that the maximum in the corresponding capacitance in Figure S17a agrees with the region at which the charge density in Figure S16 starts to saturate. In Figure S12b we can see $\varepsilon = 5.6\varepsilon_0$ that the capacitance is slightly lower in the range of bias at which the confined water region is charged. This can be linked to the effect of this latter.

Changes in t_w, they are depicted in Figures S18 and S19.

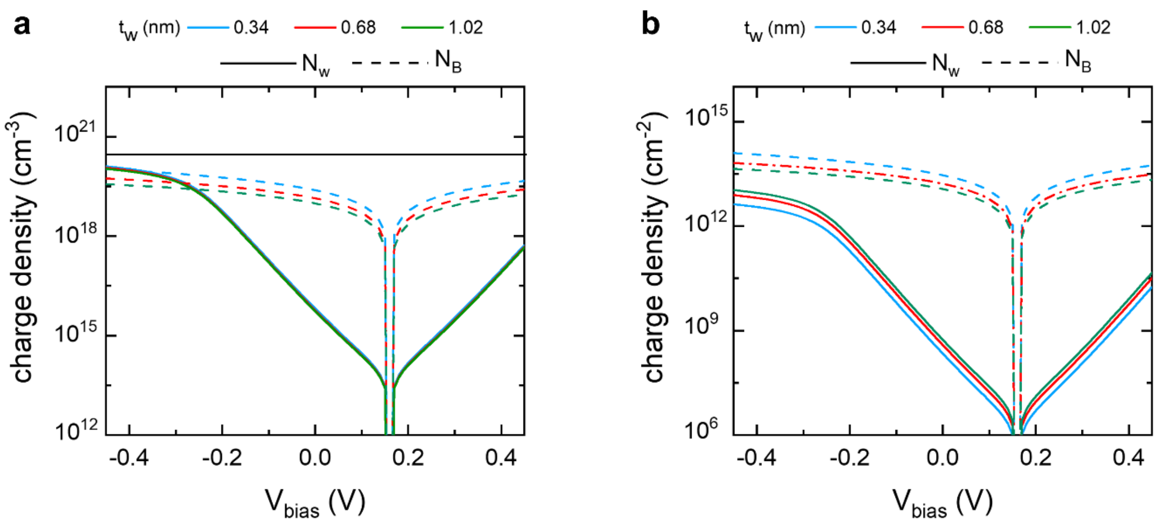

Figure S18: (a) Charge density profiles at the confined water region (N_w) and bulk electrolyte (N_B) as a function of the bias for different thicknesses. Black line indicates the limit defined by steric effects. (b) Surface charge density profiles, i.e. the thickness of the regions is taken into account, as a function of V_b.

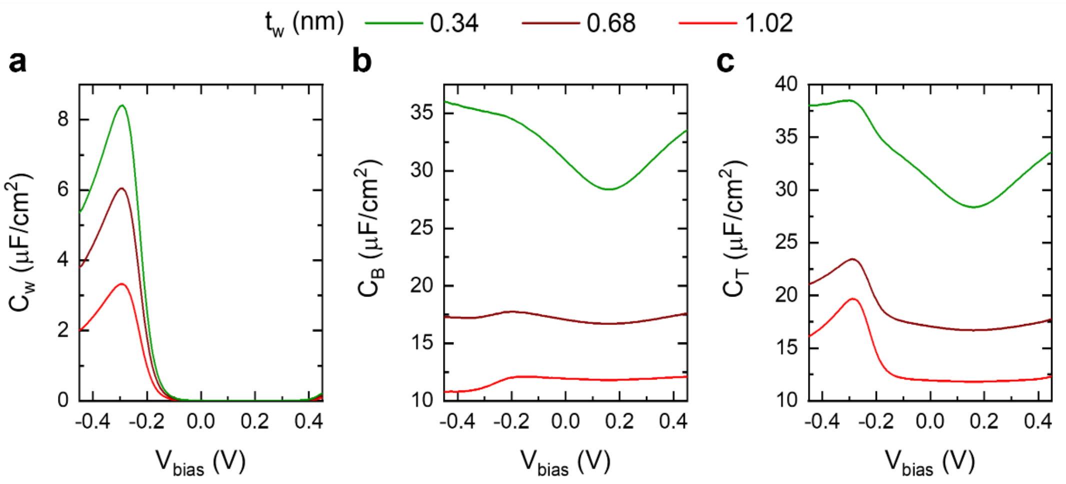

Figure S19: (a,b) Capacitances between the ITO substrate and each of the regions of the structure and (c) total capacitance of the structure.

When approaching V_b = -0.3V, the confined water region starts to accumulate enough charge to screen the ITO from the point of the bulk electrolyte. This give rise to a large accumulation of charge near the ITO interface, which is translated in the maximum depicted by C_{ITO} around this bias. When going to more negative values the confined water region cannot accumulate more charge due steric effects, so the capacitance starts to decrease. There, the main charge contribution is the one of the bulk electrolyte, but it is less coupled with the ITO due to the

presence of the charged confined water region. As the permittivity of the confined water region is increased, the maximum generated by the confined water regions is less pronounced, while the magnitude of the capacitance is increased. This is due to the better coupling between the ITO and the bulk electrolyte. In this case the contribution of the bulk electrolyte to the net charge is higher, reducing the impact of the screening of the charge of the confined water region. That is why for the highest values of permittivity we observe the expected capacitance of the electrolyte, which is ambipolar, but with a lowering below V_b = -0.3V due to the saturation of the confined water region. In this case, we observe the same behaviour for the charge density associated to the confined water region. The profiles do not depict changes with t_w, so the surface charge density is obtained in Figure S18b. There we can see how a thicker region provides a larger surface charge density, agreeing with the capacitance profiles depicted in Figure S19a. The profiles in Figure S18b show the aforementioned lowering of C_B in the range $V_b < -0.2$V. In this case it is much clearer, and it is easier to see its relation with the confined water region: the larger the magnitude of C_w, the larger the reduction in C_B.

References

(1) Drieschner, S.; Guimerà, A.; Cortadella, R. G.; Viana, D.; Makrygiannis, E.; Blaschke, B. M.; Vieten, J.; Garrido, J. A. Frequency Response of Electrolyte-Gated Graphene Electrodes and Transistors. *J. Phys. D: Appl. Phys.* **2017**, *50* (9), 095304. https://doi.org/10.1088/1361-6463/aa5443.

(2) Coy Diaz, H.; Addou, R.; Batzill, M. Interface Properties of CVD Grown Graphene Transferred onto MoS $_2$ (0001). *Nanoscale* **2014**, *6* (2), 1071–1078. https://doi.org/10.1039/C3NR03692H.

(3) Benedek, G.; Manson, J. R.; Miret-Artés, S. The Electron–Phonon Coupling Constant for Single-Layer Graphene on Metal Substrates Determined from He Atom Scattering. *Phys. Chem. Chem. Phys.* **2021**, *23* (13), 7575–7585. https://doi.org/10.1039/D0CP04729E.

(4) Toral-Lopez, A.; Marin, E. G.; Gonzalez-Medina, J. M.; Romero, F. J.; Ruiz, F. G.; Morales, D. P.; Rodriguez, N.; Godoy, A. Assessment of Three Electrolyte–Molecule Electrostatic Interaction Models for 2D Material Based BioFETs. *Nanoscale Adv.* **2019**, *1* (3), 1077–1085. https://doi.org/10.1039/C8NA00109J.

(5) Toral-Lopez, A.; Kokh, D. B.; Marin, E. G.; Wade, R. C.; Godoy, A. Graphene BioFET Sensors for SARS-CoV-2 Detection: A Multiscale Simulation Approach. *Nanoscale Adv.* **2022**, *4* (14), 3065–3072. https://doi.org/10.1039/D2NA00357K.

(6) Schwierz, N.; Horinek, D.; Netz, R. R. Reversed Anionic Hofmeister Series: The Interplay of Surface Charge and Surface Polarity. *Langmuir* **2010**, *26* (10), 7370–7379. https://doi.org/10.1021/la904397v.
